# The role of a novel decision aid to support informed decision making process in patients with a symptomatic non - lower pole renal stone < 20 mm in diameter: a prospective randomized study

**DOI:** 10.1590/S1677-5538.IBJU.2018.0198

**Published:** 2019-01-29

**Authors:** Mehmet İlker Gökce, Cağrı Akpınar, Barış Esen, Vahid Solak, Ömer Gülpınar, Yaşar Bedük

**Affiliations:** 1 Department of Urology Ankara University School of Medicine Ankara Turkey Department of Urology, Ankara University School of Medicine, Ankara, Turkey

**Keywords:** Kidney Calculi, Disease, Lithotripsy

## Abstract

**Objectives:**

To evaluate the efficacy of a novel decision aid (DA) in improving the patients’ level of knowledge and decreasing decisional conflicts while deciding for SWL vs. RIRS in case of a symptomatic renal stone <2 cm.

**Materials and Methods:**

In this prospective randomized study patients were randomized to receive either standard informing process (group 1, n=57) or DA (group 2, n=58). Level of knowledge was assessed with a questionnaire of 10 questions before and after patient informing process. Level of decisional conflict was assessed with a previously validated scoring system. Logistic regression analysis was performed to identify factors associated with adequate level of knowledge.

**Results:**

Level of knowledge increased significantly in both groups after patient informing process. The increase was significantly more prominent in group 2 (p=0.045). Percentage of patients with adequate knowledge was also higher in group 2 (56.1%vs.74.1%, p=0.04). Mean decisional conflict scale score (higher score indicates higher decisional conflict level) was also significantly higher in group1 (14.7±14.5 vs. 10.1±13.7, p=0.045). Multivariate logistic regression analysis revealed higher education level (college degree) and use of DA as factors associated with adequate level of knowledge.

**Conclusions:**

In the current study, The DA was shown to have a positive impact on level of knowledge and diminish the level of decisional conflict for patients with a symptomatic non-lower pole renal stone <20 mm. We recommend development and use of DAs for particular clinic scenarios to aid in education of patients and shared decision making process in stone disease clinics.

## INTRODUCTION

Stone disease is reported to have a prevalence of 8.8% in the United States and this prevalence also has a tendency increase (1). Careful evaluation and appropriate management of stone disease is crucial considering its short and long term effects on patient’s quality of life and renal functions.

Both shock wave lithotripsy (SWL) and retrograde intrarenal surgery (RIRS) are recommended for the management of non - lower pole renal stones < 20 mm in diameter by the most recent AUA and EAU guidelines (2, 3). The AUA guidelines emphasize the importance of shared decision making for this particular patient group (2).

The decision making process for management of stone disease relies on factors influenced by either the patient or the physician. In a recent study, Sarkissian et al. evaluated the factors that affect patient’s preferences on choosing treatment options for management of an asymptomatic renal calculi. An important finding of this study was 56.4% of patients deferred the decision of the treatment approach to the physician (4). On the other hand, in another study, the management behaviors of urologists for a lower pole stone was investigated with a web based survey and 81.2% of the participants responded that patient’s preferences were important for decision making. Therefore, involvement of patient in the decision making process should be facilitated and appropriate tools for patient education are required for this purpose (5).

Decision aids (DAs) are tools designed to educate patients on treatment options and possible outcomes. DAs have been used by the urologists especially for screening and management of prostate cancer (6, 7). We recently developed the first DA in the era of stone disease for decision making in treatment of symptomatic non - lower pole renal stones < 20 mm in diameter (8). The aim of this study is to evaluate the efficacy of this DA in improving the patient’s level of knowledge and decreasing decisional conflicts in comparison with standard patient informing process in a prospective randomized manner.

## MATERIALS AND METHODS

**Figure 1:**
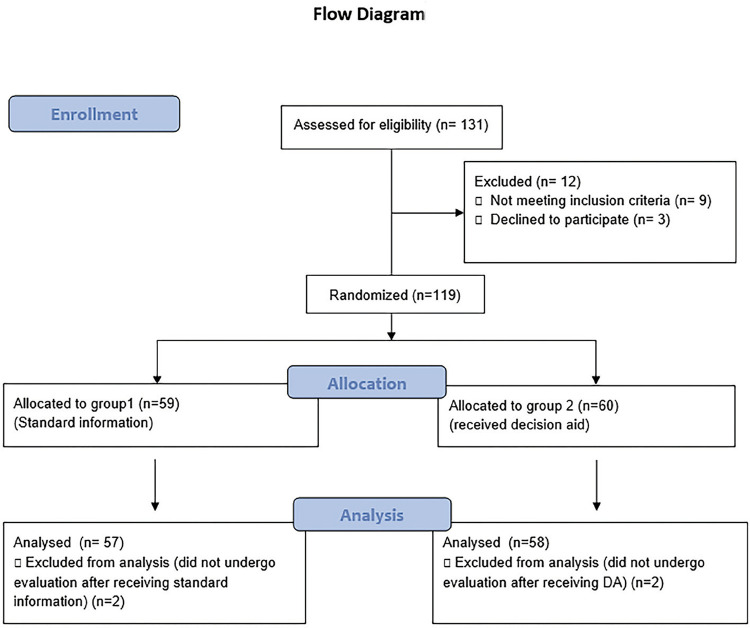
Flow diagram for enrollment of the patients to the study.

The study was approved by our Institutional Review Board (approval number: 08-428-17). In this single center randomized study, patients with symptomatic non - lower pole renal stones < 20 mm in diameter were included. Patients were included from our stone disease outpatient clinics between December 2016 and May 2017. The CONSORT statements were followed and a flow diagram is provided in Figure-1.

### Inclusion criteria

1. Patients between 18-75 years of age
2. The patient should have the ability to read and write
3. The patient should have a symptomatic non - lower pole renal stones < 20 mm and planned intervention
4. The patients should consent to be included in the study

### Interventions and data collection

The patients were randomized to two study groups. A computer software was used to generate random allocation sequence. The random allocation sequence was placed in preset numbered envelopes and a nurse opened the envelopes for each patient to perform randomization. The patients in group 1 received standard patient informing process. The information was provided verbally and included general information about stone disease, success and complication rates of SWL and RIRS, and advantages and disadvantages of these two techniques. All patients in this group were informed by a single physician. The patients in group 2 received the DA. The DA has been developed in accordance with the criteria of International Patient Decision Aid Standards (IPDAS) Collaboration and has been published recently (the DA is uploaded as supplementary material) (8).

All patients received a questionnaire of 10 questions (provided as supplementary material) to evaluate the level of knowledge on stone disease before receiving standard informing process or the DA. The questionnaire was provided again after receiving the informing process or DA to evaluate the change in the level of knowledge. The patients with correct answers for at least 8 of the 10 questions were accepted as having adequate level of knowledge.

After the informing processes, the subjects in both groups were asked about their decision to undergo SWL or RIRS. Additionally, they were asked to complete a ten question Decisional Conflict Scale which assessed uncertainty, whether subjects felt informed, had clarity on the information, and felt supported (9). The Decisional Conflict Scale provides a score between 0 and100. The higher score indicates higher level of decisional conflict and a score of ≤ 25 was accepted as having a low decisional conflict score.

The parameters collected included age, gender, level of education, history of stone disease episodes, and history of previous interventions. The primary end point was the comparison of the change in level of knowledge in both groups and comparison of decisional conflict scores. The secondary end point was the evaluation of factors that have effect on having adequate level of knowledge.

### Sample size calculation

In order to calculate the sample size, as there are no previous studies on this topic, a pilot study was conducted with 40 patients and 20 patients were provided with standard informing process and the other 20 given the DA without randomization.

After receiving the informing process, 11 of 20 (55%) patients and 16 of the 20 (80%) patients were found to have adequate level of knowledge respectively and when these values were used for effect size, at least 54 patients were required for each arm to provide a power of 80% with a significance level of 5%.

### Statistical analysis

Statistical analysis was performed with SPSS ver. 20.0 (IBM Corp. Released 2011.IBM SPSS Statistics for Windows, Version 20.0. Armonk, NY: IBM Corp.). Patient characteristics were summarized using mean ± SD or median with range for continuous variables depending on normal distribution and frequency (percentage) for categorical variables. Wilcoxon Signed-Ranks test was used to compare the level of knowledge before and after the informing process for the two groups. The Chi-square test was used to compare the categorical variables and t test or Mann-Whitney U test were used to compare the continuous variables in both groups. The percentages of patients with adequate level of knowledge and low decisional conflict scale score were also compared with Chi-square test. Univariate and multivariate logistic regression analysis was performed to identify factors associated with adequate level of knowledge (≥ 8 / 10 correct answers) after patient informing process. For statistical significance, p value of 0.05 was accepted.

## RESULTS

The number of patients randomized to group 1 and group 2 were 59 and 60 respectively. Two patients in each group did not undergo the evaluation after informing processes and data of 57 and 58 patients were analyzed. The groups were similar for age, gender, level of education and previous history of stone disease and interventions. The results are summarized in Table-1.

**Table 1:** Demographic characteristics and stone disease related history of the patient groups.

Parameters	Group 1 (n=57)	Group 2 (n=58)	P value
Age, mean±SD	46.5±5.8	46.2±5.9	0.88
**Gender, n(%)**			**0.50**
Male	33 (58.9)	30 (62.5)	
Female	24 (41.1)	28 (37.5)	
**Education level, n(%)**			**0.65**
Elementary School	12 (21.0)	15 (25.9)	
High school	25 (43.9)	27 (46.6)	
College degree	20 (35.1)	16 (27.5)	
Previous history of stone disease, n(%)	16 (28.1)	20 (34.4)	0.45
Previous history of intervention, n(%)	10 (17.5)	9 (15.5)	0.77

### Results of level of knowledge

Median (range) number of correct answers was 4 (1-9) before informing processes for both groups and significantly increased to 6 (3-10) and 8 (5-10) in group 1 (P = 0.03) and group 2 (p = 0.009) respectively. The median number of correct answers after informing process was significantly higher in group 2 compared to group 1 (6 (3-10) vs. 8 (5-10), p = 0.045). In group 1, the number of patients with adequate knowledge was 8 (14%) before informing process and significantly increased to 32 (56.1%) after informing process (p < 0.0001). Similarly, number of patients with adequate level of knowledge increased significantly after receiving the DA in group 2 (10 (17.2%) vs. 43 (74.1%), p < 0.0001)). Also, the number of patients with adequate knowledge after informing process was significantly higher in group 2 compared to group 1 (32 (56.1%) vs. 43 (74.1%), p = 0.04)).

### Results of patient’s decisions and decisional conflict scale

After the patient informing process, 20 of 57 (35.1%) patients of the patients in group 1 and 28 of the 58 (48.3%) of the patients in group 2 decided to undergo SWL (p = 0.15). The mean decisional conflict scale score of group 1 was significantly higher compared to group 2 (14.7 ± 14.5 vs. 10.1 ± 13.7, p = 0.045). When the groups were compared for percentages of patients with high decisional conflict (decisional conflict scale score > 25) significantly higher number of patients were detected to have high decisional conflict level. The results are summarized in Table-2.

**Table 2:** Comparison of the two groups for Decisional Conflict Scale scores.

Decisional Conflict Scale score	Group 1 (n=57)	Group 2 (n=58)	P value
**Total score, n (%)**			0.04
≤25	38 (66.7)	48 (82.8)	
>25	19 (33.3)	10 (17.2)	
**Uncertainty subscale, n (%)**			0.032
≤25	44 (77.2)	49 (84.5)	
>25	13 (22.8)	9 (15.5)	
I**nformed subscale, n (%)**			0.03
≤25	37 (64.9)	48 (82.8)	
>25	20 (35.1)	10 (17.2)	
**Values clarity subscale, n (%)**			0.17
≤25	40 (70.2)	47 (81.1)	
>25	17 (29.8)	11 (18.9)	
**Support subscale, n (%)**			0.25
≤25	41 (71.9)	47 (81.1)	
>25	16 (28.1)	11 (18.9)	

The univariate logistic regression analysis revealed education level (college degree), previous history of stone disease, and patient informing method (use of DA) as factors associated with having adequate level of knowledge. The results of univariate analysis are summarized in Table-3. These factors are further evaluated in a multivariate model and education level (OR: 1.88, 95%CI: 1.44-3.78, p = 0.03) and use of DA for patient informing (OR: 2.24, 95%CI: 1.80-4.12, p = 0.01) were identified as independent predictors of establishing adequate level of knowledge. History of stone disease was not identified as an independent factor associated with adequate level of knowledge in the multivariate analysis (OR: 1.18, 95%CI: 0.89-1.68, p = 0.17).

**Table 3:** Univariate logistic regression models for having adequate level of knowledge. The outcome variable is whether or not correctly answered at least 8 out of 10 questions (yes vs. no).

Parameters	OR	95% CI	p-value
Age	1.04	0.88 - 1.15	0.40
Gender	1.12	0.77 - 2.15	0.46
Education level (college degree vs. lower)	2.13	1.56 – 4.45	0.01
History of stone disease	1.73	1.22 - 2.43	0.03
History of intervention	1.15	0.87 - 2.53	0.34
Use of DA of patient informing	2.71	1.84 - 5.42	0.008

## DISCUSSION

Shared decision making is quite important in modern medicine and active participation of patients in the decision making process is mandatory. In order to achieve this goal, the patients should have adequate level of knowledge about their condition and the possible treatment modalities. We recently developed a DA for informing patients with a symptomatic non - lower pole renal stone and in this prospective randomized study the DA was found to be beneficial to establish adequate level of knowledge and lower decisional conflict when compared with standard patient informing process.

SWL and RIRS are the two treatment modalities suggested by the EAU and AUA guidelines for management of non - lower pole renal stones < 20 mm (2, 3). The patients should understand the unique advantages and disadvantages of these conditions and participate in the shared decision making process by taking into account their personal needs. The index patient 7 in the most recent AUA guidelines presents a case of a non - lower pole renal stone < 20 mm and the final conclusion is that the decision should rely on a shared decision-making approach (2).

The decision making process of the patients has been of interest. In the study by Sarkissian et al., patients admitted to a stone clinic were provided with a hypothetical scenario of asymptomatic lower pole renal stone. The patients were suggested to select one of three options: observation, SWL, and ureteroscopy. Although the scenario in that study is quite different from the target population in the current study, the main conclusion of the study was that patients mainly rely on the physicians’ choices. This result definitely emphasizes the importance of patient education (4).

Patient education on the medical conditions and benefits and limitations of the treatment options are crucial during the shared decision making process. However, this strongly depends on personal factors and standardization of this process will prevent personal bias. The physicians may have very heavy workloads and due to restricted time spent for each patient, patient education may be inadequate. DAs have potential to have benefit not only for the patients but also for the physicians and nurses as well, due to the fact that patients may be well informed before the informed consent process and ready to ask questions about the treatment choices. The DAs have potential to cover this problem as well. A Cochrane review on the use of DAs has been published in 2014 and proved the role of DAs in improving patient’s level of knowledge on treatment options, and reduce the level of decisional conflict (10).

The shared decision making process has also importance from the physicians’ point of view as well. In a recent study, a web based survey was conducted among urologists to investigate their choices for a small asymptomatic lower pole stone. A very important finding of this study was 81.2% of the urologists mentioned that patient’s preferences are one of the two most important factors for their recommendation together with concomitant calyx dilation (5). Therefore, in order to maintain a good balance between the advantages of treatment options and patient’s personal needs, standardized informing process is crucial.

We evaluated the factors that have effect on establishing adequate level of knowledge and identified patient’s level of education (a college degree) and use of the DA as associated factor in the multivariate analysis. DAs were shown to increase the level of knowledge and facilitate the informed decision making process even in the under-educated populations (6). Therefore, the benefits of using DAs in stone clinics has potential to be more prominent in the under-educated patients.

The primary end point of the study was to evaluate the role of DA in increasing patient’s level of knowledge and level of decisional conflict. The most important drawback of the study is the questionnaire used to evaluate level of knowledge is prepared for this study and is not validated. However, this questionnaire is prepared by taking into account the questions commonly asked by patients and during the processing of the questionnaire, 3 urologists collaborated to identify the important points. The second outcome parameter - decisional conflict scale - has already been validated with its scoring system (9). Another important limitation of the study is that the DA was prepared with the contribution of urologists from a single department. This may have effect on the content of the DA and therefore DAs prepared in a multi-institutional manner would be of greater value. Also, the perception of the DA may differ among patients from different socioeconomic and educational levels.

## CONCLUSIONS

Patient education to have a sufficient level of knowledge on the treatment choices is important for a successful shared decision making process. In the current study, the DA was shown to have a positive impact on level of knowledge and diminish the level of decisional conflict for patients with a symptomatic non-lower pole renal stone < 20 mm. We recommend development and use of DAs for particular clinic scenarios to aid in education of patients and shared decision making process in stone clinics, especially in case the patient has to intend for a treatment modality.
